# Supplementary material for: Asynchronous magnetic resonance elastography: Shear wave speed reconstruction using noise correlation of incoherent waves
Source: Magn Reson Med. 2022 Oct 27;89(3):990–1001. doi: 10.1002/mrm.29502 (PMC9792433; doi:10.1002/mrm.29502)
Supplement: Supplementary file 1 — DATA S1 MATLAB code used to generate the numerical simulations of Figure 1 [file MRM-89-990-s002.zip › k-Wave/helpfiles/k-wave_function_ref_cat.html]

Functions - By Category (k-Wave)


# Functions — By Category

 View Functions Alphabetically

## Contents

- Time Domain Wave Propagation in Fluid Media
- Single Frequency Wave Propagation in Fluid Media
- Time Domain Wave Propagation in Elastic Media
- Time Domain Heat Diffusion
- Reference Solutions
- Photoacoustic Image Reconstruction
- Geometry and Shape Creation
- Acoustic Absorption Coefficient Calculation and Conversion
- Material Properties
- Grid and Matrix Utilities
- Filtering and Spectral Utilities
- Display and Visualisation
- Signal Creation and Processing
- HDF5 Utilities
- System Parameters and Utilities

## Time Domain Wave Propagation in Fluid Media

|  |  |
| --- | --- |
| `angularSpectrum` | Project time-domain input plane using the angular spectrum method. |
| `calculateMassSource` | Compute k-Wave input plane from measured time-varying data. |
| `kspaceFirstOrder1D` | 1D time-domain simulation of wave propagation |
| `kspaceFirstOrder2D` | 2D time-domain simulation of wave propagation |
| `kspaceFirstOrder2DC` | 2D time-domain simulation of wave propagation using C++ code |
| `kspaceFirstOrder2DG` | 2D time-domain simulation of wave propagation on a GPU using C++ CUDA code |
| `kspaceFirstOrder3D` | 3D time-domain simulation of wave propagation |
| `kspaceFirstOrder3DC` | 3D time-domain simulation of wave propagation using C++ code |
| `kspaceFirstOrder3DG` | 3D time-domain simulation of wave propagation on a GPU using C++ CUDA code |
| `kspaceFirstOrderAS` | Axisymmetric time-domain simulation of wave propagation |
| `kspaceFirstOrderASC` | Axisymmetric time-domain simulation of wave propagation using C++ code |
| `kspaceSecondOrder` | Fast time-domain simulation of wave propagation for homogeneous media |

## Single Frequency Wave Propagation in Fluid Media

|  |  |
| --- | --- |
| `angularSpectrumCW` | Project CW input plane using the angular spectrum method. |
| `calculateMassSourceCW` | Compute k-Wave input plane from measured CW data. |
| `acousticFieldPropagator` | Calculate acoustic field for CW source. |
| `acousticFieldPropagatorC` | Calculate acoustic field for CW source using C++ code. |

## Time Domain Wave Propagation in Elastic Media

|  |  |
| --- | --- |
| `pstdElastic2D` | 2D time-domain simulation of elastic wave propagation |
| `pstdElastic3D` | 3D time-domain simulation of elastic wave propagation |

## Time Domain Heat Diffusion

|  |  |
| --- | --- |
| `bioheatExact` | Compute exact solution to Pennes' bioheat equation in homogeneous media |
| `kWaveDiffusion` | Time-domain simulation of heat diffusion and perfusion |

## Reference Solutions

|  |  |
| --- | --- |
| `focusedBowlONeil` | Compute O'Neil's solution for focused bowl transducer |
| `mendousse` | Compute Mendousse's solution for nonlinear wave propagation in viscous media |

## Photoacoustic Image Reconstruction

|  |  |
| --- | --- |
| `kspaceLineRecon` | 2D linear FFT reconstruction |
| `kspacePlaneRecon` | 3D planar FFT reconstruction |

See also kspaceFirstOrder1D, kspaceFirstOrder2D, and kspaceFirstOrder3D for time-reversal image reconstruction

## Geometry and Shape Creation

|  |  |
| --- | --- |
| `makeArc` | Create a binary map of an arc within a 2D grid |
| `makeBall` | Create a binary map of a filled ball within a 3D grid |
| `makeBowl` | Create a binary map of a bowl within a 3D grid |
| `makeCartCircle` | Create a 2D Cartesian circle or arc |
| `makeCartSphere` | Create a 3D Cartesian sphere |
| `makeCircle` | Create a binary map of a circle within a 2D grid |
| `makeDisc` | Create a binary map of a filled disc within a 2D grid |
| `makeLine` | Create a binary map of a straight line within a 2D grid |
| `makeMultiArc` | Create a binary map of multiple arcs within a 2D grid |
| `makeMultiBowl` | Create a binary map of multiple bowls within a 3D grid |
| `makeSphere` | Create a binary map of a sphere within a 3D grid |
| `makeSphericalSection` | Create a binary map of a sphere segment within a 3D grid |

## Acoustic Absorption Coefficient Calculation and Conversion

|  |  |
| --- | --- |
| `attenComp` | Attenuation compensation using time-variant filtering |
| `db2neper` | Convert decibels to nepers |
| `fitPowerLawParams` | Fit power law absorption parameters for highly absorbing media |
| `neper2db` | Convert nepers to decibels |
| `powerLawKramersKronig` | Calculate dispersion for power law absorption |
| `waterAbsorption` | Calculate ultrasound absorption in distilled water |

## Material Properties

|  |  |
| --- | --- |
| `waterAbsorption` | Calculate ultrasound absorption in distilled water |
| `waterDensity` | Calculate density of air-saturated water with temperature |
| `waterNonlinearity` | Calculate B/A of water with temperature |
| `waterSoundSpeed` | Calculate the sound speed in distilled water with temperature |

## Grid and Matrix Utilities

|  |  |
| --- | --- |
| `cart2grid` | Interpolate a set of Cartesian points onto a binary grid |
| `expandMatrix` | Enlarge a matrix by extending the edge values |
| `findClosest` | Return the closest value in a matrix |
| `fourierShift` | Resample data using a Fourier interpolant |
| `getSpacedPoints` | Create vector of log or linear spaced points |
| `getOptimalPMLSize` | Find PML size to give the smallest prime factors |
| `grid2cart` | Return the Cartesian coordinates of the non-zero points of a binary grid |
| `interpCartData` | Interpolate data from a Cartesian to a binary sensor mask |
| `interpftn` | Resample data using Fourier interpolation |
| `kWaveGrid` | Class definition for k-Wave grid |
| `loadImage` | Load an image file |
| `maxND` | Return the value and indices of the largest value in an N-D array |
| `minND` | Return the value and indices of the smallest value in an N-D array |
| `numDim` | Return the number of matrix dimensions |
| `resize` | Resize a matrix |
| `reorderBinarySensorData` | Reorder data from a binary sensor mask |
| `reorderSensorData` | Reorder sensor data from kspaceFirstOrder2D based on angle |
| `revolve2D` | Form 3D matrix from revolution of 2D matrix |
| `roundEven` | Round towards the nearest even number |
| `roundOdd` | Round towards the nearest odd number |
| `unmaskSensorData` | Reorder data recorded using a binary sensor mask |

## Filtering and Spectral Utilities

|  |  |
| --- | --- |
| `applyFilter` | Filter input with low, high, or band pass filter |
| `envelopeDetection` | Extract signal envelope using the Hilbert Transform |
| `filterTimeSeries` | Filter signal using the Kaiser windowing method |
| `gaussianFilter` | Filter signals using a frequency domain Gaussian filter |
| `getAlphaFilter` | Create filter for `medium.alpha_filter` |
| `getBLI` | Compute underlying Fourier band-limited interpolant (BLI) |
| `getFDMatrix` | Create a matrix of finite-difference coefficients |
| `getWin` | Return a frequency domain windowing function |
| `gradientFD` | Calculate the gradient using a finite-difference method |
| `gradientSpect` | Calculate the gradient using a Fourier spectral method |
| `sharpness` | Calculate image sharpness metric |
| `spect` | Compute the single sided amplitude and phase spectrums |
| `smooth` | Smooth a matrix |
| `vesselFilter` | Frangi's 3D vessel filter |

## Display and Visualisation

|  |  |
| --- | --- |
| `beamPlot` | Plot volumetric data using intersecting planes |
| `flyThrough` | Display a three-dimensional matrix slice by slice |
| `getColorMap` | Return default k-Wave color map |
| `overlayPlot` | Overlay two images |
| `saveTiffStack` | Save volume data as a tiff stack |
| `scaleFig` | Resize current figure window |
| `stackedPlot` | Stacked linear plot |
| `voxelPlot` | 3D plot of voxels in a binary matrix |

## Signal Creation and Processing

|  |  |
| --- | --- |
| `addNoise` | Add Gaussian noise to a signal for a given SNR |
| `createCWSignals` | Generate array of CW signals from amplitude and phase |
| `envelopeDetection` | Extract signal envelope using the Hilbert Transform |
| `extractAmpPhase` | Extract amplitude and phase from CW signals |
| `focus` | Create input signal based on source mask and focus position |
| `fwhm` | Compute the full width at half maximum |
| `gaussian` | Create a Gaussian distribution |
| `gaussianFilter` | Filter signals using a frequency domain Gaussian filter |
| `hounsfield2density` | Convert Hounsfield units to density |
| `kWaveTransducer` | Class definition for k-Wave linear array transducer |
| `logCompression` | Log compress an input signal |
| `scanConversion` | Convert scan-lines in polar coordinates to a B-mode ultrasound image |
| `toneBurst` | Create an enveloped single frequency tone burst |

## HDF5 Utilities

|  |  |
| --- | --- |
| `h5compare` | Compare the contents of two HDF5 files |
| `writeAttributes` | Write attributes to a k-Wave HDF5 file |
| `writeFlags` | Write input flags to a k-Wave HDF5 file |
| `writeGrid` | Write grid and PML properties to a k-Wave HDF5 file |
| `writeMatrix` | Write MATLAB matrix to a k-Wave HDF5 file |

## System Parameters and Utilities

|  |  |
| --- | --- |
| `benchmark` | Run performance benchmark |
| `checkFactors` | Return the maximum prime factor for a range of numbers |
| `checkStability` | Return maximum stable time step for k-space fluid models |
| `getDateString` | Create a string of the current date and time |
| `getComputerInfo` | Return information about computer and k-Wave version |
| `getkWavePath` | Return pathname to the k-Wave Toolbox |
| `scaleSI` | Scale a number to nearest SI unit prefix |
| `scaleTime` | Convert seconds to hours, minutes, and seconds |
